# Temporomandibular joints disorders (TMDs) prevalence and their relation to anxiety in dental students

**DOI:** 10.12688/f1000research.76178.1

**Published:** 2022-03-03

**Authors:** Lujain Homeida, Emtenan Felemban, Wed Kassar, Mazen Ameen, Salwa Aldahlawi

**Affiliations:** 1 Basic and Clinical Oral Sciences,, Umm-Al-Qura University, Makkah, 24382, Saudi Arabia

**Keywords:** TMD, DENTAL, STUDENTS, PAIN, STRESS, PARAFUNCTIONAL HABITS

## Abstract

**Background**: Temporomandibular joint disorders (TMDs) are very common disorders affecting the population and causing pain. Researchers have reported a high prevalence of TMDs among university students due to increased distress. The aims of this study were to determine the frequency of TMDs in Umm al-Qura University (UQU) dental students using the Diagnostic Criteria for Temporomandibular Disorders (DC/TMD), and to examine the relationship between anxiety, bruxism, and TMDs among those students.

**Methods**: The DC/TMD pain screener questionnaire was completed by dental students. Students who reported TMD pain or had at least one positive TMD symptom were invited to the dental clinic for a full TMJ evaluation. History of bruxism was documented and an ultra-brief tool for detecting anxiety and depression (Patient Health Questionnaire-4 PHQ) was completed by all students.

**Results**: A total of 240 students responded to the TMD pain screener in which 119 reported at least one TMJ symptom. Only 93 dental students presented to clinical examination in which 64.5% (n=60) of them had temporomandibular joint disorders. Disc displacement with reduction and local myalgia (38.7% & 32.25%, respectively) were the most frequent diagnosis. A total of 29% (n=27) of students had more than one diagnosis. Overall, 41 participants (44.09%) reported a positive response to the anxiety scale and (n=38) 40% of participants reported parafunctional habits. Both the history of bruxism and anxiety were significantly related to TMDs (P=0.0002) and also significantly higher in women of higher academic years (P≤0.01).

**Conclusions**: This study found a high prevalence of TMDs among UQU dental students. Disc displacement with reduction was the most prevalent disorder. Bruxism and anxiety were associated with painful TMDs.

## Introduction

Temporomandibular joint disorders (TMDs) are a very common group of musculoskeletal disorders affecting the temporomandibular joint (TMJ) and the face causing pain. They are considered a significant public health burden in approximately 5% to 12% of the general population ( National Institute of Dental and Craniofacial Research 2018, July). Painful TMD has a direct impact on the person’s quality of life and daily activity ( Schiffman, Ohrbach *et al*. 2014). The TMD has a multifactorial pathogenesis in which it involves physiological and/or psychological factors like emotional distresses. Chronic parafunctional habits can cause repetitive trauma to the masticatory system, which may result in painful TMD episodes ( Schiffman, Ohrbach *et al*. 2014). Parafunctional habits including but not limited to bruxing and clenching are known to have a critical role in aggravation and progression of TMD ( Chisnoiu, Picos *et al*. 2015). Furthermore, psychosocial distress is also considered an important comorbidity contributing to TMD ( Schiffman, Ohrbach *et al*. 2014). Some individuals, when exposed to stressful situations, tend to activate the stomatognathic system by clenching or grinding their teeth and increasing masticatory muscle contraction in order to relieve their stress. This increased masseter activation during stress and decrease in a relaxing situation was highly associated with the presence of TMD in individuals under more stress ( Calixtre, Gruninger *et al*. 2014).

Many studies have looked into the psychological stress among university students and its impact on student’s academic achievement and well-being. High prevalence of mental issues between university students was reported ( Adlaf, Gliksman *et al*. 2001). Stallman *et al* evaluated mental stress among Australian universities students and found a high prevalence of mental health problems (19.2 %) and subsyndromal symptoms (67.4%) which were significantly higher than those of the general population ( Stallman 2010). This supports that university student population live under more stress than the general population. Thus, the prevalence of TMDs is relatively high among university students of different specialties.

Binoleil *et al*, assessed the prevalence of headaches and painful TMDs and examined the relationship between TMDs, headaches, and depression rates among dental and medical students. They reported higher depression scores in patients with painful TMD compared to patients without TMD ( Benoliel, Sela *et al*. 2011). Furthermore, the relationship between stress level and painful TMD in students of health science was supported in few more studies locally ( Alkhudhairy, Al Ramel *et al*. 2018) and internationally where stress played an important role in TMD progression ( Monteiro, Zuim *et al*. 2011, Wieckiewicz, Grychowska *et al*. 2014).

The Research Diagnostic Criteria for Temporomandibular Disorders (RDC/TMD) was first proposed in 1992 and has been used widely as diagnostic protocol for TMD research ( Dworkin and LeResche 1992). However, more research was done over the years to improve its validity and clinical utility. In 2014, an evidence-based new Diagnostic Criteria for Temporomandibular Disorders (DC/TMD) was published and was considered a valid and reliable screening tool for use in clinical and research settings and included important modifications to the original RDC/TMD ( Schiffman, Ohrbach *et al*. 2014). An acceptable sensitivity and specificity for a definitive diagnosis are considered as sensitivity ≥ 70% and specificity ≥ 95%. DC/TMD has diagnostic algorithms used to diagnose the most common pain-related TMD and most common intra-articular disorders ( Schiffman, Ohrbach *et al*. 2014). The Axis I diagnostic algorithm consists of two parts; a self-report instrument where it is used for pain screening. The second part is used for TMJ clinical examination. The DC/TMD Axis II protocol included instruments to evaluate pain behavior, psychological status, and psychosocial functioning ( Schiffman, Ohrbach *et al*. 2014).

To the best of our knowledge, the prevalence of TMDs among Umm al-Qura University (UQU) dental students has not been evaluated. In this study, we aimed to determine the prevalence of TMDs in UQU dental students using Diagnostic Criteria for Temporomandibular Disorders (DC/TMD). Also, to examine the relation between anxiety, self-reported bruxism and TMDs among UQU dental students.

## Methods

This cross-sectional study was approved from Umm Al-Qura University (UQU), College of Dentistry Institutional Review Board (IRB) # (98-18). This study took place at collage of Dental Medicine Umm al Qura University. Data were collected between July 2019 and December 2019.

### Screening dental students for TMDs

The UQU dental collage program starts at 2 ^nd^ level and it is involves 5 academic years followed by internship year. All UQU dental students (year 2 - internship year) were included in this study and were invited by email to fill The Research Diagnostic Criteria (RDC) three-items pain screener on TMD symptoms ( Schiffman, Ohrbach *et al*. 2014). Non-dental students from UQU and dental student from other universities were excluded. All participants have given their consent to participate in the study. Demographic data were collected including age, gender, marital status and year of study. In addition, history of TMD diagnosis and history of parafunctional habits (bruxism), and the use of chronic medication was included. The response to each of the three questions was documented.

### TMJ clinical examination and diagnosis

The respondents who reported TMD pain or had at least one positive answer to one of the 3-items questionnaire were invited to have a full TMJ clinical examination in the specialty clinic at UQU teaching hospital. The TMJ clinical examination included detailed assessment of the TMJ position and structure, range of motion measurements, and palpation of muscle of mastication following DC/TMD protocol. The clinical examination provided to all students was performed by one oral medicine/TMD specialist. Written informed consent was obtained from all participants prior to clinical examination.

The DC/TMD diagnostic criteria algorithms were followed to reach a TMD diagnosis. This included pain-related temporomandibular disorders (local myalgia, myofascial pain, myofascial pain with referral, arthralgia, and headache attributed to TMD) and intra-articular disorders (disc displacement with reduction, disc displacement with reduction with intermittent locking, disc displacement without reduction with limited opening, disc displacement without reduction without limited opening). History of bruxism was obtained and those who had painful TMD were referred to an oral medicine specialist clinic for further treatment.

### Anxiety and depression scale

During the clinical examination, a valid and ultra-brief tool for detecting anxiety and depression (Patient Health Questionnaire-4 PHQ) was completed by all participants ( Kroenke, Spitzer *et al*. 2009). This four-items questionnaire (PHQ-4) consisted of two core anxiety and two core depression items. The total score of this scale ranges from 0-12 and categorized as normal (0 –2), mild (3–5), moderate (6 – 8), and severe (9–12). This instrument is not diagnostic, however, is indicator for further assessment of possible clinical disorder warranting treatment.

### Data analysis

The data analysis was performed using Statistical Package for the Social Sciences version 22 (SPSS Inc., Chicago, IL, USA, RRID:SCR_019096). Student T-test and chi-square analysis were used to relate the existence of TMD problem to age, gender, academic year, history of bruxism and anxiety level. Also, to compare the anxiety scores among male and female groups. Statistical significance was set at *P* ≤ 0.05.

## Results

A total of 304 electronic questionnaires were sent via email to all dental students at Umm al-Qura university who were between 2 ^nd^ year to the internship with a total of 6 academic years. A total of 240 questionnaires were completed and returned with a compliance rate of 78.9% ( Homeida 2022). The demographics of respondents from the pain screening questionnaire can be found in Table 1.

**Table 1.** Demographic data of participants who responded positive to the pain screener and who presented for the temporomandibular joint (TMJ) clinical examination.

	Respondents to pain screener (n=240)	Participants in TMJ clinical examination (n=93)
**Age**	22.38 ± 1.2 year	22.3 ± 1.25 year
**Gender**		
Male	131 (54%)	45 (48.4%)
Female	109 (46%)	48 (51.6%)
**Academic Year**		
2 ^nd^ year	11 (4.5%)	3 (3.2%)
3 ^rd^ year	46 (19.1%)	12 (12.9%)
4 ^th^ year	42 (17.5%)	14 (15.1%)
5 ^th^ year	56 (23.3%)	25 (26.9%)
6 ^th^ year	55 (22.9%)	27 (28%)
Internship year	30 (12.5%)	12 (14%)

### Temporomandibular joint disorder pain screener results

Out of the 240 responders, 49.5% (n=119) reported either TMD pain or other TMD symptoms in the past 30 days. And 20.4% (n=49) reported TMJ pain that comes and goes. The majority of students 30% (n=72) reported jaw habits with 25% (n=60) had pain on opening, 16% (n=40) reported pain on chewing hard or tough food and 14% (n=35) had pain with jaw activities such as talking and yawning ( Table 2).

**Table 2.** Results of temporomandibular joint disorder pain screener (n=240).

	Frequency *n* (%)
In the last 30 days, how long did any pain last in your jaw or temple area on either side?	
No pain	189 (78.7)
Pain comes and goes	49 (20.4)
Pain is always present	2 (0.8)
In the last 30 days, have you had pain or stiffness in your jaw on awakening?	
Yes	45 (18.7)
No	195 (81.2)
last 30 days, did the following activities change any pain (that is, make it better or it worse) in your jaw or temple area on either side? Chewing hard or tough food	
Yes	40 (16)
No	200 (83)
Opening your mouth or moving your jaw forward or to the side	
Yes	60 (25)
No	180 (75)
Jaw habits such as holding teeth together, clenching, grinding, or chewing gum	
Yes	72 (30)
No	168 (70)
Other jaw activities such as talking, kissing, or yawning	
Yes	35(14)
No	205 (86)

### Prevalence of TMDs among dental students

All the responders who reported TMD pain or symptoms (n=119) were invited for a TMJ examination. A total of 93 (78%) subjects presented for clinical assessment. The demographics of the subjects received the clinical examination are presented in Table 1. During TMJ clinical examination, more than half of the subjects 64.5% (n=60) had temporomandibular disorders (TMDs), while only 35.4% (n=33) students had normal TMJ findings at the time of the examination.

**Figure 1.**
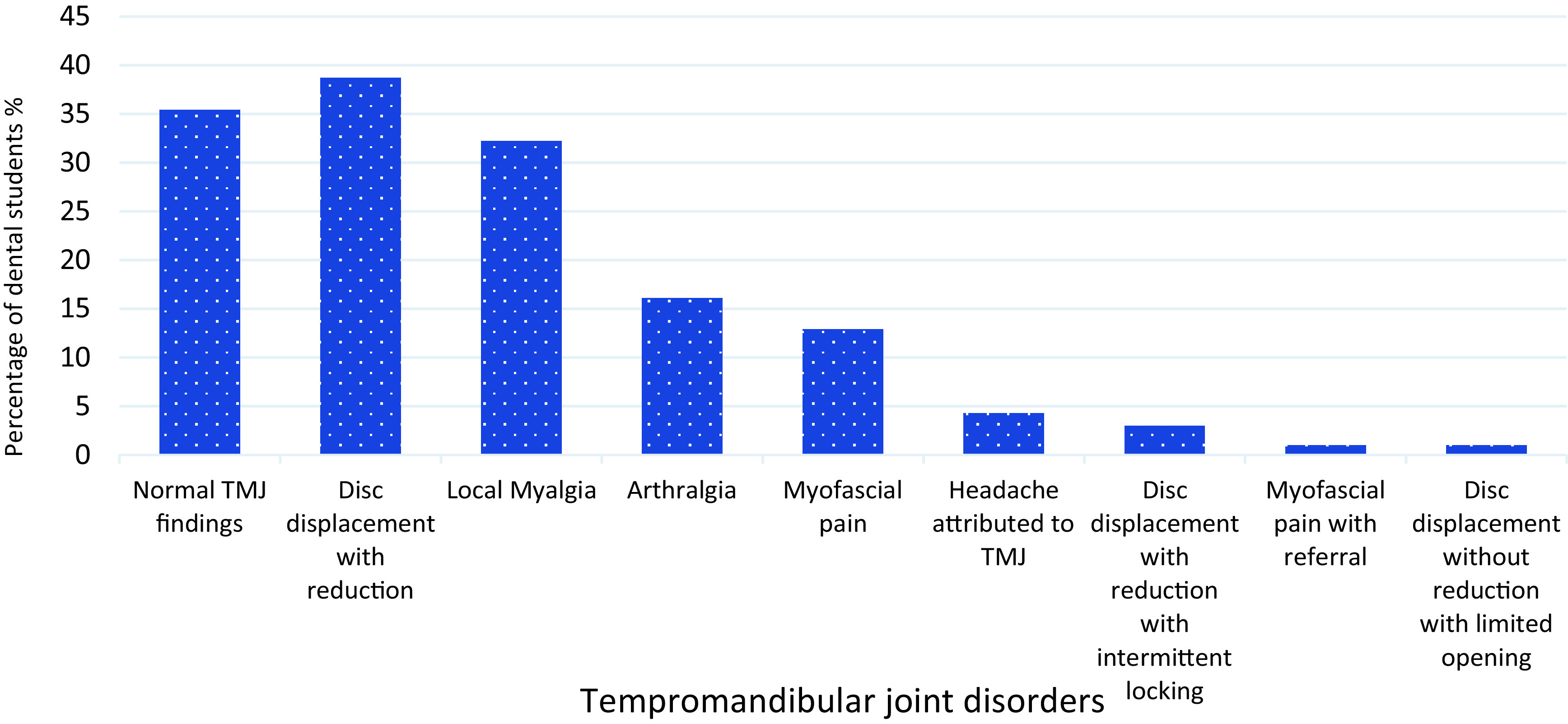
Percentage of dental students presenting with DC/TMD Diagnosis. DC/TMD: Diagnostic Criteria for Temporomandibular Disorders.

Disc displacement with reduction was the most frequent diagnosis 38.7%(n=36) followed by local myalgia 32.25% (n=30) and arthralgia 16.1% (n=15) ( Figure 1). Interestingly, 29% (n=27) students had more than one diagnosis and coexistence of disc displacement with reduction and local myalgia was found to be the most frequent combination in 19.3% (n=18) students ( Table 3). Overall, the diagnosis of TMD was significantly higher in the female students compared to the male students (P≤0.022). Both, pain-related disorders and intra-articular disorders were significantly higher in females with (P=0.027) and (P=0.024), respectively. Also, disc displacement with reduction showed a significant increase with the higher academic year (P≤0.01).

**Table 3.** Gender based distribution of TMDs, Anxiety scale and history of bruxism.

	Female N (%)	Male N (%)	Total N (%)
**Total number of participants**	45 (48.3)	48 (51.6)	93 (100)
**Bruxism**	28 (30)	10 (10.7)	38 (40.8)
**TMDs**			
*Normal TMJ findings*	12 (12.9)	21 (22.5)	33 (35.4)
*Disc displacement with reduction*	22 (23.6)	14 (15)	38.7 (36)
*Local myalgia*	16 (17)	14 (15)	32.2 (30)
*Myofascial pain*	11 (11.8)	1 (1)	12.9 (12)
*Arthralgia*	5 (5.3)	10 (10.7)	16.1 (15)
*Headache attributed to TMD*	4 (4)	0	4.3 (4)
*Disc displacement with reduction with intermittent locking*	3 (3)	0	3 (3)
*Myofascial pain with referral*	1 (1)	0	1 (1)
*Disc displacement without reduction with limited opening*	1 (1)	0	1 (1)
**PHQ-4**			
*Mild*	16 (17)	4 (4)	20 (21.5)
*Moderate to severe*	20 (21.5)	1 (1)	21 (22.5)

TMD: Temporomandibular disorders. PHQ-4: Patient Health Questionnaire-4.

### Anxiety and depression scale and history of parafunctional habits

In total, 41 participants (44.09%) reported a positive response to the anxiety and depression scale. In which, 48.7% (n=20) had a mild score and 51% (n=21) had moderate to severe anxiety score ( Table 3). Of the 41 subjects with a positive response to PHQ-4, 31 (73%) had been diagnosed with a painful TMDs. Moderate to severe anxiety was significantly associated with TMDs (P=0.006).

Overall, 40% of the participants (n=38) reported a parafunctional habit. Out of 38 (71%) with a history of bruxism, 27 had TMDs. Self-reported history of bruxism was significantly associated with TMDs in all students (P≤0.01).

**Figure 2.**
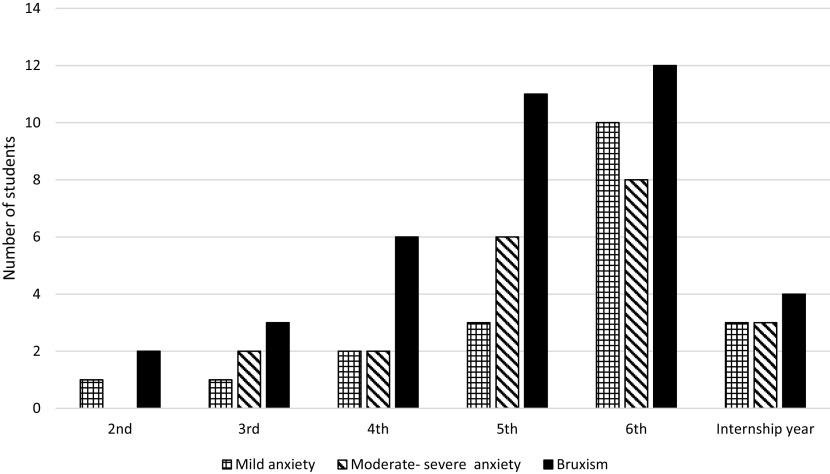
History of bruxism and anxiety levels significantly increased with higher academic years and declined in internship year.

Both the history of bruxism and the level of anxiety were significantly related (P=0.0002) and also significantly higher in females than males (P≤0.01). Besides, the anxiety level and history of bruxism significantly increased with higher academic years (P≤0.05). Those high scores of anxieties and bruxism were reversed in the internship year ( Figure 2).

## Discussion

The present study found that half of UQU dental students reported at least one TMD symptom. Moreover, the clinical examination found that 64% were diagnosed with at least one TMJ disorder and about 30% (one- third) had multiple diagnoses. Bruxism and high anxiety levels were related to TMDs in this student population.

Evidence suggests that TMD is a common complain among students. The prevalence of TMD reported by this study is higher than what has been reported by other studies in Saudi universities which ranged between 25-39% ( Alkhudhairy, Al Ramel *et al*. 2018, Srivastava, Shrivastava *et al*. 2021). Studies that addressed general university Saudi students also reported TMD prevalence of 20 to 50% ( Zulqarnain, Khan *et al*. 1998, Habib, Al Rifaiy *et al*. 2015, Zwiri and Al-Omiri 2016, Srivastava, Shrivastava *et al*. 2021). All the above-mentioned studies with the exception of Srivastava *et al.* relied on self-administered questioners to identify subjects with TMDs and did not include TMJ clinical examination to confirm the diagnosis as the present study which may explain the difference in prevalence. When RDC/TMD algorithm was used to estimate the prevalence of TMD, 30 to 36% of dental students were found to have TMD ( Fernandes Azevedo, Camara-Souza *et al*. 2018, Lövgren, Österlund *et al*. 2018). The present study used RDC/TMD pain screener to identify the subject with possible TMJ symptoms first, then, only those with positive responses to the pain screener were examined clinically for TMD diagnosis and that may explain the higher prevalence of TMDs.

Disc displacement with reduction was the most prevalent TMD disorder followed by myalgia. This finding concurs with a systematic review in which disc displacement of TMJ was highly prevalent TMD in the general population with prevalence ranging from 18 to 35% ( Naeije, Te Veldhuis *et al*. 2013). Also, the finding of the increasing prevalence of disc displacement with age in this study is in alignment with other studies were disc displacement with reduction develops during childhood and adolescence and it’s prevalence levels off towards adulthood ( Marpaung, van Selms *et al*. 2019, Sankuratri, Verma *et al*. 2021). Although myalgia was the reported as the commonest diagnosed condition in some studies ( Srivastava, Shrivastava *et al*. 2021), it was the second most prevalence condition in this study. On the other hand, females had a higher prevalence of TMDs compared to males. This findings was in alignment with other studies ( Bagis, Ayaz *et al*. 2012, Naeije, Te Veldhuis *et al*. 2013, Wieckiewicz, Grychowska *et al*. 2014, de Melo Junior, Aroucha *et al*. 2019, Xie, Lin *et al*. 2019, Sankuratri, Verma *et al*. 2021, Srivastava, Shrivastava *et al*. 2021). Interestingly, 30% of the subjects had more than one TMDs diagnosis. Similar finding were reported by Azevedo 2018 *et al* ( Fernandes Azevedo, Camara-Souza *et al*. 2018) which highlights the importance of diagnosis and early management of TMDs.

Previous cross-sectional studies reported a significant increase in anxiety and depression scores among medical and dental students in different Saudi universities ( Inam 2007, Aboalshamat, Hou *et al*. 2015, Basudan, Binanzan *et al*. 2017). In this study, 44% of the students with TMD symptoms reported a positive response to the PHQ-4 with half of them classified as having moderate to severe anxiety. Female dental students had a higher PHQ-4 mean score than male students which was in agreement with similar studies done on Saudi dental students ( Inam 2007, Al-Saleh, Al-Madi *et al*. 2010, Benoliel, Sela *et al*. 2011, Al-Sowygh 2013). Overall, the rate of anxiety in women has been reported to be higher than men ( Kessler, Sonnega *et al*. 1995, Steel, Marnane *et al*. 2014, Xie, Lin *et al*. 2019). This could be explained by the slower processing in neurotransmitter serotonin which has a critical role in anxiety and depression. Besides, women are more sensitive to specific hormone such as corticotropin-releasing factor which has an important role in stress response ( Bangasser, Curtis *et al*. 2010).

The majority of those students who showed moderate to severe levels of anxiety and depression were diagnosed with painful TMDs on clinical examination and therefore, anxiety was found to be related to TMDs. Other studies have conflicting results. stress and anxiety were positively associated with TMD in university students in general and dental students in particular ( Ton, Mota *et al*. 2020, Jaiswal and Deshpande 2021). While Azevedo *et al* found no association between anxiety and TMD ( Fernandes Azevedo, Camara-Souza *et al*. 2018). Anxiety and stress in dental students can be caused by many external factors like exams, clinical requirements and academic assignments. This study was conducted during the academic year which could contribute to the higher prevalence of anxiety and TMDs.

In general, oral parafunctional habits are known as a major contributor to TMDs and play an important role in its progression ( Chisnoiu, Picos *et al*. 2015). In the present study, self-reported bruxism was significantly associated with TMDs. This finding concurs similar findings in Swedish dental students in which participants with TMD reported significantly higher oral parafunctional habits ( Lövgren, Österlund *et al*. 2018). Moreover, the results of this study are in accordance with Jaiswal *et al*, where they reported significant relationship between TMDs and parafunctional habits in Indian dental students ( Jaiswal and Deshpande 2021).

History of bruxism and anxiety levels were found to be higher among the senior dental students at UQU. Similar findings were reported by other studies in which a statistical significant relationship between anxiety and the para-functional habit was revealed ( Paterson, Lamb *et al*. 1995, Ahlberg, Lobbezoo *et al*. 2013). Most individuals who have anxiety disorders tend to relieve their stress by clenching and/or grinding their teeth and contracting masticatory muscle which leads to activation of the stomatognathic system ( Calixtre, Gruninger *et al*. 2014). This increase in stress levels among final-years students can be due to more participants from those two academic years. However, it can be also explained by the higher clinical demands and more workload during these final clinical years. Declining of both bruxism and anxiety parameters was noted in participants from the internship year where there is a significant decrease in academic load and clinical requirements. Similar findings were reported by Ahuja ( Ahuja, Ranjan *et al*. 2018).

## Limitation of the study

This is a cross-sectional study in which only association between variables is detected. Longitudinal studies are needed to prove causation. The absence of a control group for anxiety screening was regarded as a limitation for this study, as no clinical examination nor depression score was done for the participants who denied TMD pain or symptoms. Also, a comparison of TMDs prevalence with age-matched general population (non-dental students) was not considered in this study and it is recommended for future research projects.

## Conclusions

TMDs is highly prevalent among dental students. Disc displacement with reduction was the most predominant one. Greater prevalence was observed among females and higher academic years. Bruxism and anxiety were associated with painful TMDs.

## Data availability

### Underlying data

Dryad: Temporomandibular Joints Disorders TMDs Prevalence and Its Relation to Anxiety in Dental Students. https://doi.org/10.5061/dryad.kkwh70s62 ( Homeida 2022).

This project contains the following underlying data:

- Pain screener results for 240 participants.xlsx
- TMD diagnosis results of 93 participants.xlsx

Data are available under the terms of the Creative Commons Zero “No rights reserved” data waiver (CC0 1.0 Public domain dedication).
