# Supplementary material for: Temporomandibular joints disorders (TMDs) prevalence and their relation to anxiety in dental students
Source: F1000Res. 2022 Apr 27;11:271. Originally published 2022 Mar 3. [Version 2] doi: 10.12688/f1000research.76178.2 (PMC10643880; doi:10.12688/f1000research.76178.2)
Supplement: Peer review history

# Reviewer report: Reviewer response for version 2

**DOI:** 10.5256/f1000research.133454.r176706

**Published:** 2023-11-13

**Authors:** Mahnaz Fatahzadeh

**Affiliations:** 1 Department of Diagnostic Sciences, Rutgers School of Dental Medicine, Newark, NJ, USA

This study is interesting and pertinent. Authors provide a concise background and explain the reason for performing this study, describe their scientific approach and adequately define inclusion and exclusion criteria. Data analysis seems reasonable. Although the study or its findings are not novel, the results are interesting and generally in line with previous reports. Authors explain their findings in a focused and clear manner in the discussion section.

 Here are a few points to consider:

 Title: The study population were dental students in UQU but it is not stated in the title.

 In the results section of abstract, line 7: state what is meant by participants (students completing the initial survey or those who were examined). 

 Page 4, 1st paragraph: Since this study specifically investigates the prevalence of TMD in dental students in UQU, there is no need to mention dental students from other universities were excluded.

 Please rephrase legend for clarity. For example, Table 1:  Demographic of participants with positive response to the pain screener and those receiving temporomandibular joint (TMJ) clinical examination.

 Please rephrase legend for figure 2 for clarity. For  example, Figure 2; Distribution of self-reported history of bruxism and anxiety levels according to academic year of dental training among study participants

 There are minor grammatical issues which could be easily addressed by editing and proof reading.

Is the work clearly and accurately presented and does it cite the current literature?

Yes

If applicable, is the statistical analysis and its interpretation appropriate?

Yes

Are all the source data underlying the results available to ensure full reproducibility?

Yes

Is the study design appropriate and is the work technically sound?

Yes

Are the conclusions drawn adequately supported by the results?

Yes

Are sufficient details of methods and analysis provided to allow replication by others?

Yes

Reviewer Expertise:

Oral Medicine

I confirm that I have read this submission and believe that I have an appropriate level of expertise to confirm that it is of an acceptable scientific standard.

# Reviewer report: Reviewer response for version 2

**DOI:** 10.5256/f1000research.133454.r136082

**Published:** 2022-05-04

**Authors:** Arwa Farag

**Affiliations:** 1 Division of Oral Medicine, Department of Diagnostic Sciences, Tufts School of Dental Medicine, Boston, MD, USA

The suggestions I provided in my previous review were addressed precisely.

Is the work clearly and accurately presented and does it cite the current literature?

Yes

If applicable, is the statistical analysis and its interpretation appropriate?

I cannot comment. A qualified statistician is required.

Are all the source data underlying the results available to ensure full reproducibility?

Yes

Is the study design appropriate and is the work technically sound?

Yes

Are the conclusions drawn adequately supported by the results?

Yes

Are sufficient details of methods and analysis provided to allow replication by others?

Yes

Reviewer Expertise:

NA

I confirm that I have read this submission and believe that I have an appropriate level of expertise to confirm that it is of an acceptable scientific standard.

# Reviewer report: Reviewer response for version 1

**DOI:** 10.5256/f1000research.80143.r126222

**Published:** 2022-04-07

**Authors:** Arwa Farag

**Affiliations:** 1 Division of Oral Medicine, Department of Diagnostic Sciences, Tufts School of Dental Medicine, Boston, MD, USA

Dear author,

 Thank you for conducting this investigation. The study aimed to estimate the prevalence of TMDs among dental student and characterize the most common TMD diagnoses along with associated symptoms/conditions. The methodology followed was optimal, starting from screening to comprehensive clinical assessment and self-reported questionnaires. Recruitment and inclusion of the study participants were done using the gold standard diagnostic criteria (RDC-TMD). All the utilized tools of assessment were reliable and previously validated for TMD population.

 Below, I’m including minor points/comments, that I hope will further optimize this well-put-together manuscript.

 Methodology:

 In the exclusion criteria, author should specify that health history was obtained and those with underlying rheumatoid conditions, generalized pain symptoms, connective tissue disorders were excluded from the study.

 Discussion:

 It would great if the author can provide some statistics/citations for prevalence of TMD (disc displacement/myalgia) along with stress and anxiety in the general population and compare them to this special population (dental students). This will provide insight, and clearly highlight, the increased prevalence of these conditions among dental students.

 Thank you once  again for allowing me the opportunity to review this work.

Is the work clearly and accurately presented and does it cite the current literature?

Yes

If applicable, is the statistical analysis and its interpretation appropriate?

I cannot comment. A qualified statistician is required.

Are all the source data underlying the results available to ensure full reproducibility?

Yes

Is the study design appropriate and is the work technically sound?

Yes

Are the conclusions drawn adequately supported by the results?

Yes

Are sufficient details of methods and analysis provided to allow replication by others?

Yes

Reviewer Expertise:

OFP and OM

I confirm that I have read this submission and believe that I have an appropriate level of expertise to confirm that it is of an acceptable scientific standard, however I have significant reservations, as outlined above.

# Response

**Published:** 2022-04-21

**Authors:** Lujain Homeida

**Affiliations:** Umm al-Qura University, College of Dentistry, Saudi Arabia

Response to TMD reviewer comments

 Thank you for the comments and the review, below are our responses

**1 - i** **n the exclusion criteria, author should specify that health history was obtained and those with underlying rheumatoid conditions, generalized pain symptoms, connective tissue disorders were excluded from the study**.

 Thank you for the comment, the material and method section was amended as follows:

 under TMJ clinical examination and diagnosis the following sentence was added:

 “Detailed medical history was obtained and subjects with underlying rheumatoid conditions, generalized pain symptoms, connective tissue disorders were excluded from the study”.

 2- **It would great if the author can provide some statistics/citations for prevalence of TMD (disc displacement/myalgia) along with stress and anxiety in the general population and compare them to this special population (dental students). This will provide insight, and clearly highlight, the increased prevalence of these conditions among dental students**.

 Thank you for this point. Most of the literature on TMD addressed specific population (patients, medical or dental students, or university students) very few literature addressed general population or included non TMD general population as a comparison group.

 The following paragraph was added to the discussion.

 'In comparison to the general population, TMD prevalence ranges between 5 – 35 % (Schiffman, Ohrbach et al. 2014, Nadershah 2019). However, none of those studies reported on the association of TMD with stress, anxiety or phycological factors in general population. In general,  anxiety disordered have been reported in 35 % of chronic pain patients compared to only 18 % of the general population (Poleshuck, Bair et al. 2009). in addition, Reissmann et al. compared patients diagnosed with TMD and general population without TMD related pain and reported that Trait anxiety is significantly associated with diagnoses of TMD pain. One point increase in the State-Trait Anxiety Inventory score related to an increase of the odds for pain-related TMD by the factor 1.04  (Reissmann, John et al. 2014).  A similar finding reported by kmeid et al. were TMDs was  significantly associated with depression, anxiety, and stress among Lebanese population (Kmeid, Nacouzi et al. 2020)'
